# Editorial: Molecular Physiology in Molluscs

**DOI:** 10.3389/fphys.2019.01131

**Published:** 2019-09-06

**Authors:** Xiaotong Wang, Youji Wang

**Affiliations:** ^1^School of Agriculture, Ludong University, Yantai, China; ^2^College of Fisheries and Life Science, Shanghai Ocean University, Shanghai, China

**Keywords:** mollusc, physiological process, morphological character, molecular mechanism, interaction with other factors

Molluscs are second only to arthropods in terms of the number of living animal species and are the largest marine phylum, comprising ~23% of all named marine organisms. Many molluscs have economic importance. Much progress has been made in the study of molluscan physiology, with two Nobel Prizes having been awarded for research on molluscan physiology (neurophysiology). Researching the giant axon of the squid, Professors A. L. Hodgkin and A. F. Huxley revealed the mechanisms involved in nerve impulses and, as a result, were awarded the Nobel Prize for Physiology or Medicine in 1963 (Wilbur and Yonge, 1964). Professor Eric Kandel, sharing the Nobel Prize for Physiology or Medicine 2000, found that, as snails learned, chemical signals changed the structure of the connections between cells (synapses), where the signals are sent and received, indicating that short- and long-term memories are formed by different signals (Kandel, 2005). Despite these prominent results, many obstacles remain in molluscan physiology research, such as the short domestication time, observation difficulties, absence of appropriate instruments and model animals, particularly the lack of a proliferative cell line. The embryonic cell line developed from the snail *Biomphalaria glabrata* (Bge) is the only existing cell line originating from a molluscan species (Yoshino et al., 2013). Compared with aquatic vertebrates, such as fish, the study of the functional validation of physiology-related genes has lagged behind in molluscs. However, with the development of molecular biology and omics techniques, molluscan physiology research has made significant gains over the past few years. The editorial office of Frontiers suggested that we should bring together related developments from other researchers, and publish a Research Topic on “molecular physiology in molluscs” in Frontiers in Physiology. At last, all 19 papers were published in this topic. These papers, united in this Research Topic, cover a broad thematic range and mainly focus on the molecular mechanisms of molluscan physiology.

In recent years, ocean acidification (OA), caused by further increases in atmospheric CO_2_ partial pressure (Diaz et al., 2018), has become a hot Research Topic. Given that the main component of the external shells of mollusc (Marin et al., 2008) or internal bones (Marschner et al., 2013) or statoliths (Kaplan et al., 2013) is calcium carbonate, molluscs might be sensitive to ocean acidification (Parker et al., 2013; Wang et al., 2017; Campanati et al., 2018). Cao et al. reveal that OA reduced the resistance of Pacific oyster *Crassostrea gigas* to *Vibrio splendidus* challenge because of the increased energy expenditure, whereas Liao et al. report the energy metabolism and antioxidant responses of the Yesso scallop (*Patinopecten yessoensis*) under OA, reporting the tissue-specific effects related to energy metabolism. However, Kriefall et al. had different opinions about the physiological responses on seawater acidification because they found that far future OA levels have observable, but not severe, impacts on *Crepidula fornicata* larvae.

Cardiac-related physiology of molluscs is an interesting subject. Chen et al. show different transcriptomic responses to thermal stress in heat-tolerant and heat-sensitive Pacific abalones indicated by cardiac performance, providing insight into the different heat-response strategies used by these animals. Cardiac indices were a novel approach used to investigate the scallop, *Chlamys farreri*. In their study, Xing et al. demonstrate that heart rate is a good indicator of thermal limit, whereas heart amplitude might indicate optimal growth temperature, and the rate-amplitude product could be an index of myocardial oxygen consumption, which provides basic information on the factors that might impact cardiac performance in scallops.

Via byssal threads, bivalves, such as mussels and scallops, can adhere to a variety of hard substrates under wet conditions (Amini et al., 2017; Waite, 2017). However, byssus adhesion is also an important cause of aquatic biofouling. In their paper,  Li et al. focused on molecular bases of adhesive mechanisms of byssus in the highly invasive fouling mussel *Limnoperna fortunei*; their results will be of use to develop strategies against biofouling by freshwater organisms. Zhang et al. characterize an atypical metalloproteinase inhibitor-like protein (Sbp8-1) from the byssus of *C. farreri*, suggesting that Sbp8-1 might act as a crosslinker in the scallop byssus. This study also provides inspiration for the design of water-resistant materials.

Salinity is a primary physical factor affecting not only the distribution and physiological metabolism of molluscs (Jones et al., 2019), but also the areas suitable for the aquaculture of marine species. Jia and Liu report the molecular characterization of full-length Na^+^/K^+^-ATPase cDNA in Pacific abalone *Haliotis discus hannai*, finding that sudden salinity changes affect *NAK* gene transcription activation, translation, and enzyme activity via a cAMP-mediated pathway. Compared with Pacific abalone, Hong Kong oyster *Crassostrea hongkongensis* has a wider range of salinity adaptation. Using *in situ* transcriptomes, Xiao et al. reveal divergent adaptive responses to hyper- and hyposalinity in *C. hongkongensis*, suggesting the adaptive plasticity of marine molluscs.

Hemocytes of molluscs are involved in not only various physiological functions, but also innate immunity (Serpentini et al., 2000; Ray et al., 2013; Wang et al., 2013). Brokordt et al. propose that the hemocyte energy metabolic capacity potentially underlies cellular and molecular immune responses in a reproduction–immunity tradeoff in the scallop *Argopecten purpuratus*. The functioning of the host immune system relies on the frequent renewal and appropriate functioning of hemocytes, but the underlying renewal mechanism remains elusive at the gene level. Zhou et al. identify a transcription factor, LIM homeobox 9 (ChLhx9), in *C. hongkongensis*, showing that it induces hemocyte apoptosis by activating apoptotic genes or pathways, providing new insights into the functioning of molluscan hemocytes.

For human beings, the accumulation of toxic substances in molluscs is a serious food safety problem (Ragi et al., 2017). However, it is also unclear how the accumulation of toxic substances in molluscs will affect their own physiological activities, and how these effects might be impacted by other environmental factors. Cao et al. carried out a comparative *in vivo* study of the toxicity of saxitoxin (STX) on *C. gigas* and *C. farreri*, identifying different biochemical and immunotoxicity biomarkers in these two bivalves. Shi et al. demonstrate that anthropogenic noise and toxicity of cadmium have synergetic effects on the feeding activity, metabolism, and ATP synthesis of the blood clam *Tegillarca granosa*, which might be caused by the addition of stress responses and neurotransmitter disturbances.

Given their economic importance, the nutrition and growth physiology of molluscs have always been a focus of molluscan aquaculture research. Li et al. identify and characterize key genes in the hedgehog pathway of *C. gigas*, analyzing their accumulation and RNA localization patterns, and showing that the hedgehog pathway could be involved in *C. gigas* myogenesis. Although important for bivalve aquaculture, no artificial diet for bivalves has been successfully developed (Knauer and Southgate, 1999). By using a gas chromatography time-of-flight mass spectrometry (GC–TOF/MS)-based metabolomics approach, Yang et al. compare the metabolomics responses of pearl oysters (*Pinctada fucata martensii*) fed a formulated diet indoors with those of oysters cultured with natural diet outdoors, finding that the formulated diet could be an appropriate substitute for a natural diet, although its nutrient levels are insufficient.

Triploid oysters have been marketed for some years (Gong et al., 2004; Kang et al., 2013). However, data on the differences in the nutritional value between diploid and triploid oysters are lacking. Qin et al. compare the biochemical composition and nutritional quality of diploid vs. triploid *C. hongkongensis*, reporting that triploid *C. hongkongensis* has a better nutritional value and taste compared with the diploid *C. hongkongensis* during the reproductive phase. Thus, the authors suggest that we should consume triploid oysters harvested during the summer breeding season.

Finally, Kong et al. identify and analyze two novel short peptidoglycan recognition proteins from the deep sea Vesicomyidae clam *Archivesica packardana*, while Chen et al. report the method of piggyBac transposon-mediated transgenesis for the first time in a mollusc (*C. gigas*). Li et al. introduce the traditional Chinese nouns of “Yin” and “Yang” into the study of molecular physiology, identifying Forkhead Box L2 (FOXL2) and Doublesex And Mab-3 Related Transcription Factor 1L (DMRT1L) as the “Yin” and “Yang” Genes for female and male gonadal differentiation, respectively, in the bivalve mollusc *Patinopecten yessoensis*. Thus, those authors proposed that molecular sex differentiation is established before morphological sex differentiation in this species.

Together, the papers forming this Research Topic highlight exciting recent achievements in the varied world of molecular physiology research in molluscs, which will positively impact our understanding of molluscan physiology and advance the aquaculture of this economically important group. In future, cell line and genome editing developments could strongly promote the further research into the molecular physiology of molluscs.

## Author Contributions

All authors listed have made a substantial, direct and intellectual contribution to the work, and approved it for publication.

### Conflict of Interest Statement

The authors declare that the research was conducted in the absence of any commercial or financial relationships that could be construed as a potential conflict of interest.
